# Draft Genome Sequence of *Marinomonas* sp. Strain D104, a Polycyclic Aromatic Hydrocarbon-Degrading Bacterium from the Deep-Sea Sediment of the Arctic Ocean

**DOI:** 10.1128/genomeA.01211-13

**Published:** 2014-01-23

**Authors:** Chunming Dong, Xiuhua Bai, Qiliang Lai, Yanrong Xie, Xin Chen, Zongze Shao

**Affiliations:** a Key Laboratory of Marine Genetic Resources, Third Institute of Oceanography, State Oceanic Administration, Xiamen, China; b State Key Laboratory Breeding Base of Marine Genetic Resources, Xiamen, China; c Key Laboratory of Marine Genetic Resources of Fujian Province, Xiamen, China; d Life Science College, Xiamen University, Xiamen, Fujian, China

## Abstract

*Marinomonas* sp. strain D104 was isolated from a polycyclic aromatic hydrocarbon-degrading consortium enriched from deep-sea sediment from the Arctic Ocean. The draft genome sequence of D104 (approximately 3.83 Mbp) contains 62 contigs and 3,576 protein-encoding genes, with a G+C content of 44.8%.

## GENOME ANNOUNCEMENT

*Marinomonas* spp. are marine bacteria of wide distribution. Up to now, 22 type strains from different species have been identified in this genus (http://www.bacterio.net/marinomonas.html). According to the NCBI genome and assembly databases, only four genome sequences of *Marinomonas* have been reported, those of two type strains, *Marinomonas mediterranea* MMB-1^T^ and *Marinomonas posidonica* IVIA-Po-181^T^ (1, 2), as well as two nontype strains, *Marinomonas* sp. strain MED121 and *Marinomonas* sp. strain MWYL1. Here, we report the genome sequence of another *Marinomonas* strain, designated *Marinomonas* sp. D104. This strain was isolated from a polycyclic aromatic hydrocarbon (PAH)-degrading consortium, which was enriched from the deep-sea subsurface sediment of the Makarov Basin (170°29′W, 87°04′N, water depth of 4,000 m) in the Arctic Ocean. Although only one primary study indicated that *Marinomonas* can mineralize PAHs (3), our previous studies showed that strain D104 can degrade a wide variety of PAHs, such as naphthalene, fluorene, phenanthrene, anthracene, and pyrene at 25°C (unpublished data). In addition, strain D104 shows the highest sequence similarity, 97.72%, to *Marinomonas ushuaiensis* DSM 15871^T^ (4), followed by another 21 type species of the genus *Marinomonas* (94.51 to 96.94% similarity). These results indicate that strain D104 is a potential new species within the genus *Marinomonas.*

Genomic DNA was purified from strain D104 with an AxyPrep bacterial genomic DNA miniprep kit (Axygen) according to the manual instructions. The genome was sequenced using the Genome Analyzer IIx system at Shanghai Majorbio Bio-pharm Technology Co., Ltd. (Shanghai, China), which produced 1,471 Mbp paired-end reads of 80 bp with an insert size of 300 bp. Approximately 1,356-Mbp high-quality reads were assembled with SOAP*denovo* version 1.05 (5). The final genome assembly has 353-fold coverage and contains 62 scaffolds composed of 49 contigs (>1,000 bp), with a total size of 3,833,369 bp, an N_50_ contig length of 207,434 bp, and a mean G+C content of 44.8%.

Gene prediction and annotation of the draft genome were carried out using the NCBI Prokaryotic Genome Annotation Pipeline (PGAP) (http://www.ncbi.nlm.nih.gov/genomes/static/Pipeline.html) (6). The draft genome is composed of 3,576 putative protein-encoding genes (2,822 with annotated functions) and contains 4 rRNA genes, 5 small noncoding RNA genes, and a total of 63 tRNA genes for all 20 amino acids. Furthermore, 27 pseudogenes were also identified in the draft genome.

According to the annotation results, many genes possibly involved in the degradation of aromatic compounds were found in the genome of *Marinomonas* sp. D104, including one aromatic ring-cleaving dioxygenase and five naphthalene 1,2-dioxygenase genes (7, 8). Moreover, three genes encoding protocatechuate 3,4-dioxygenase and five genes encoding protocatechuate 4,5-dioxygenase were also found. They carry out the oxidative cleavage of protocatechuate, which is an important intermediate metabolite during the degradation of aromatic compounds (9). In addition, one cytochrome P450 gene was present in the genome. A previous study demonstrated that cytochrome P450 is also involved in the degradation of PAHs (10). The genome sequence of *Marinomonas* sp. D104 helps to improve understanding of the PAH-degrading mechanism of *Marinomonas* spp.

### Nucleotide sequence accession number.

This whole-genome shotgun project has been deposited at DDBJ/EMBL/GenBank under the accession no. AYOZ00000000. The version described in this paper is the first version.
